# DyNamic Interactive Anticipation–Time for a Paradigmatic Shift

**DOI:** 10.1007/s40279-024-02135-9

**Published:** 2024-11-03

**Authors:** Rouwen Cañal-Bruland, David L. Mann

**Affiliations:** 1 Department for the Psychology of Human Movement and Sport, Friedrich Schiller University Jena, Seidelstraße 20, 07749 Jena, Germany; 2 Department of Human Movement Sciences, Vrije Universiteit Amsterdam, Amsterdam Movement Sciences and Institute Brain and Behavior Amsterdam (iBBA), Amsterdam, The Netherlands

## Abstract

Everyday human interactions require observers to anticipate the actions of others (e.g., when walking past another in a corridor or choosing where to hit a ground stroke in tennis). Yet, experimental paradigms that aim to examine anticipation continue to use simplistic designs that are not interactive and therefore fail to account for the real-life, social nature of these interactions. Here we propose a fundamental, paradigmatic shift toward a “dynamic interactive anticipation” paradigm that models real-life interactions. We propose that it will change the way behavioral experimentalists study anticipation and spark theory development by unravelling the mechanisms underlying anticipation in real-time interactions.

## Key Points

**Table:** 

Research on anticipation needs to fundamentally evolve to allow us to describe, explain, predict, and optimize real-life anticipatory behavior.
We propose a paradigmatic shift that will fundamentally change our current experimental approaches away from examining passive, isolated observers toward studying dynamic, interactive social encounters in real time.
Exciting new developments in AI, VR, and AR mean that genuinely interactive paradigms are now possible to realize the proposed paradigmatic shift.

## Introduction

Everyday human interactions require observers to anticipate the actions of others (e.g., when walking past another in a corridor or choosing where to hit a ground stroke in tennis). Yet, experimental paradigms that aim to examine anticipation continue to use simplistic designs that fail to truly account for the real-life, social nature of these interactions. New developments in AI (artificial intelligence), VR (virtual reality), and AR (augmented reality) mean that genuinely interactive paradigms are now possible. Here we wish to propose a fundamental, paradigmatic shift toward an “interactive anticipation” paradigm that will change the way behavioral experimentalists study anticipation and spark theory development by unravelling the mechanisms underlying anticipation in real time interactions.

To illustrate what we mean by anticipation, consider the following example: if a baseball batter initiated their motor response to a pitcher’s throwing action only after reliable visual information about ball flight became available, the batter’s motor response would in many cases be too late and they would miss the ball. This is, first and foremost, due to the latencies in processing sensory information into motor responses [1]. It is therefore that in such instances we need to anticipate the future states of the to-be-hit object, plan our motor responses in advance, and hence act under varying levels of uncertainty. To stay with the example: to hit successfully, anticipation is key.

Despite the considerable theoretical and empirical progress made in anticipation research over the last 50 years [2–4], we propose that the field is in urgent need of a paradigmatic shift. This is because paradigmatically, the focus of study has been on studying anticipation by solely looking at the anticipator (i.e., observer) in isolation, usually responding to video footage of another person on computer or life-size screens, thereby having neglected to truly embrace the social dynamics and real-time interactions between actors. To fundamentally improve our understanding of anticipatory behavior in real-life, a new paradigm is needed. In this vein, the aim of this paper is to propose a new paradigmatic approach toward “dynamic interactive anticipation” that takes into account the complex, dynamic interplay between actors in anticipatory situations. Inspired by interdisciplinary advances from the social cognitive neurosciences, in this perspective we call for joint efforts to develop and establish the interactive anticipation approach, which is based on the premise that “social cognition is fundamentally different when we are in interaction with others rather than merely observing them” [5]. To empirically test this idea, a paradigmatic shift is necessary that allows for dynamic, interactive, real-time encounters to be captured.

## Time for a Paradigmatic Shift

In the cognitive sciences, for a long time the answer to the question of how we are able to predict the action intentions of others was dominated by the view that mental states such as intentions cannot be visually perceived, as they are hidden away in the minds of other people [6]. This view was challenged by the kinematic specification of dynamics (KSD) principle [7, 8], arguing that movements specify the causes of the movements. More specifically, Runeson [7] argued that kinematics specify the dynamics of the movements, the latter referring to what causes or constrains the movements. Runeson proposed that inner mental states such as intentions play a dynamic role in generating movements. It follows that according to the KSD principle, inner mental states can be directly inferred from the very movements [7]. Today, there is compelling evidence that indeed (i) intentions are specified in the kinematics and hence available in movements and (ii) that observers can pick up this information from the kinematics to predict the behavior of others [9]. This insight has been influential in various domains, including joint action [10], the neuroscience of social cognition [11], and the development of computational frameworks for motor control and social interaction [12].

Importantly, these findings converge with one of the most robust findings in anticipation research showing that expert performers are better able to pick up and use advance kinematic information than novices [2, 4]. The initial empirical approach in this field was grounded in information processing theory and aimed at identifying the perceptual–cognitive processes underlying expert anticipation [13]. This early focus on perceptual–cognitive anticipation led to the adoption of a specific paradigmatic approach. This initial paradigm focuses on the predictions of a passive observer, for instance, a badminton player who is trying to predict where an opposing player is likely to hit the shuttle [14]. The experimental approach is based on filming representative footage of opposing players’ shots, creating videos of these situations, playing these videos to badminton players of different skill levels, and temporally occluding the videos at various moments in time to examine and show that skilled players can anticipate, for instance, shot directions earlier and better than novices [2].

This observer approach affords a high level of experimental control and convenience, but has been increasingly criticized for its typical reliance on verbal or button press responses that are not likely to be ecologically valid measures for the motor behaviors they aim to represent [15]. In support of this critique, research from cognitive psychology, the cognitive neurosciences, and several neighboring disciplines additionally showed that cognition, including anticipation, is grounded in sensorimotor coupling and hence the mutual influences of sensory information processing and motor actions, thereby supporting an embodied view of perception and cognition and advocating action-based paradigms [16, 17]. Consequently, the sensorimotor anticipation approach calls for the inclusion of ecologically valid motor responses, promoting a paradigm in which the observer (i.e., participant) is free to move and respond as in the real situation. This change certainly is a step in the right direction, as evidence from the sport sciences indeed shows that the anticipatory advantage of skilled athletes increases when responding with a natural action [18], and that gaze patterns change when movement is incorporated to more closely reflect those likely used in the performance environment [19]. This advanced paradigm remains insufficient, however, because it does not break away from the classic paradigm’s exclusive focus on the observer, and hence still does not account for the dynamic, real-time interactions between the two interacting individuals that are commonplace in natural situations. For instance, when an opponent in boxing throws a punch, as soon as the observer (the attacked boxer) initiates a motor response (e.g., swerves to miss the punch), this movement does not go unnoticed by the opponent. The opponent will perceive those initial movements of the observer and adapt their own movements, for instance, by changing their intended punch direction, or by performing feints as if to pretend to punch in one direction to generate a desired movement from the observer. Similarly, if the observer attempts themselves to strike at the opponent, then the opponent will need to alter their own movements and even see additional opportunities to attack. This results in a cascade of dynamic interactions that the currently adopted paradigms struggle to capture. However, the development of a proper theory of anticipation needs to account for these interactions and needs to be able to explain and predict the subsequent anticipatory behavior.

**Fig. 1:**
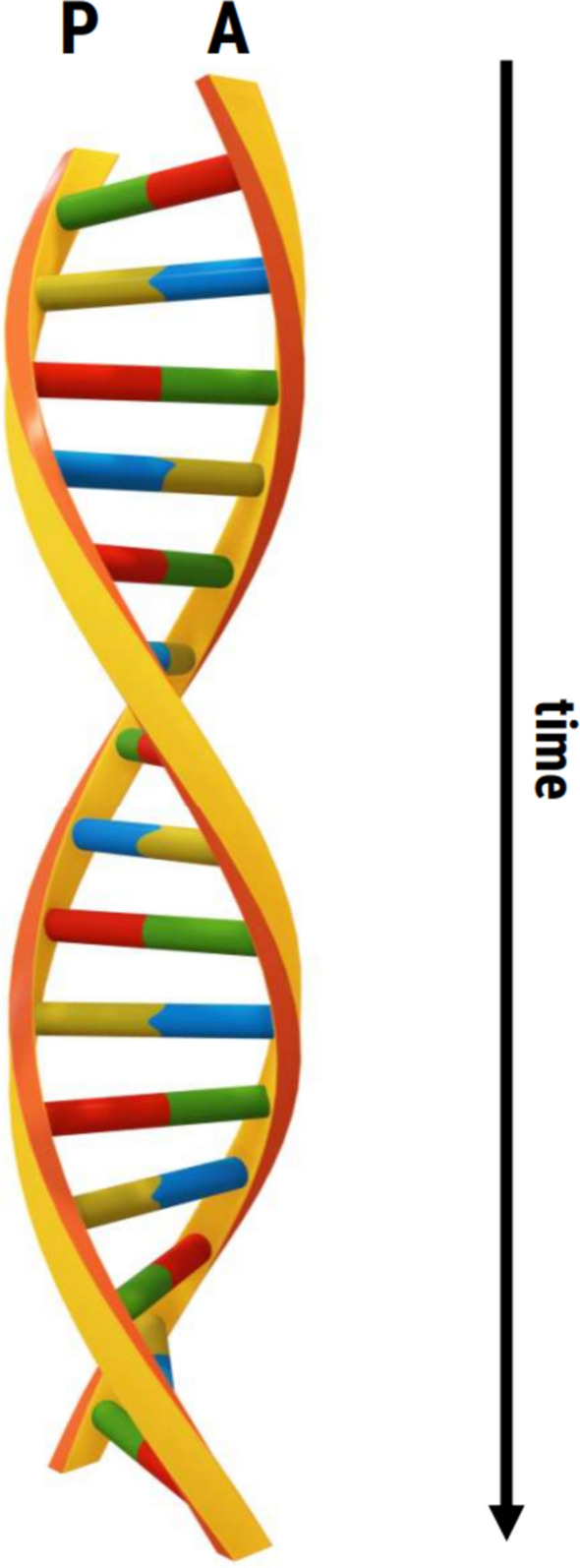
An illustration of DyNamic Interactive Anticipation (DNIA). The coiled double helix resembles the complex dynamic cascade of interactions between a participant (P) and an adversary (i.e., an avatar: A). Initially, P observes and anticipates future states of A (green part of the green–red connecting pair at the top step of the staircase) on the basis of the currently observed executed behavior of A (red part of the green–red connecting pair at the top). Subsequently, P initiates an action as a reaction to the originally anticipated, now current state of A. The next, third step indicates that A observes and anticipates future states of P on the basis of the currently observed, executed behavior of A, and so on. The dynamic cascade of interactions unfolds until the behavioral point of no return for P is reached (e.g., the goalkeeper has ultimately initiated a jump to the left in a penalty situation)

Here we argue that research on anticipation needs to fundamentally evolve to allow us to describe, explain, predict, and optimize real-life anticipatory behavior. This change is necessary and important because anticipation research—including our own—turned a blind eye to the idea that “social cognition is fundamentally different when we are in interaction with others rather than merely observing them” [5]. This premise has started to revolutionize research in the cognitive neurosciences, and it is time to similarly develop and establish a novel “dynamic interactive anticipation” approach and instigate a paradigmatic shift in the way anticipatory behavior is conceived and studied. The novel conceptual approach will integrate theoretical and empirical insights from different disciplines such as the social cognitive neurosciences, motor control, and sport psychology. The paradigmatic shift will fundamentally change our current experimental approaches away from examining passive, isolated observers toward studying dynamic, interactive social encounters in real time. Not only will the participant react to the opponent’s movements, but crucially, the opponent will dynamically adapt their movements in response to the participant’s actions, thereby initiating a complex dynamic cascade of interactions that successful anticipatory behavior needs to solve (see Fig. 1). We refer to this new paradigm as DyNamic Interactive Anticipation (DNIA).

To realize this paradigmatic shift, we should embrace and apply technologies such as AI, VR, and AR that (i) allow for full experimental control, (ii) enable participants to move and perform as in a real situation, and (iii) realistically simulate the sensory input of the real environments including actors/opponents that dynamically change their behaviors according to the real-time movements of the participant [20]. This paradigmatic shift not only is necessary to theoretically and empirically guide the field to new frontiers, but it also offers promise as a means of developing techniques and interventions that help improve anticipatory performance in many commonplace social interactions, be it in sport competitions, when driving a car in heavy traffic, or predicting a suspect’s actions as a police officer. To illustrate the urgent need and relevance for the proposed paradigmatic shift, we provide a practical example that both experts and non-experts should find easy to follow: the football penalty.

## The Football Penalty Scenario as an Example of Interactivity

The penalty scenario in football provides a prominent example of an interactive anticipatory task. In the classic penalty situation, a kicker places the ball 11 m from the goal line, and an opposing goalkeeper attempts to prevent the ball from being kicked into their goal. Early research into anticipation in football penalties required goalkeepers to view video footage of penalty takers and to anticipate the direction into which the ball would have been kicked [21, 22]. Footage was typically occluded at the moment that the kicker made contact with the ball, or at the moment(s) immediately before or after contact. Occlusion at or around contact requires the goalkeeper to anticipate the likely ball direction on the basis of largely kinematic information only. These situations were indeed not interactive: the movements of the kicker were predetermined and in no way reliant on the movements of the goalkeeper.

Evidence from the behavior of kickers in actual penalty situations shows that interactivity is in many cases vital, and therefore that the typical observer paradigm is missing an important element of expertise. While in some situations a kicker can take a goalkeeper-independent strategy whereby they will kick in a particular direction irrespective of the movements of the goalkeeper, in other situations a kicker will use a goalkeeper-dependent strategy whereby the direction in which they kick the ball will depend largely on any early movements made by the goalkeeper [23, 24]. Those kickers do not necessarily have a predetermined direction in which they intend to kick the ball; instead, in their approach to the ball, they seek to anticipate the direction in which the goalkeeper will move, and subsequently attempt to kick the ball in a different direction. That is to say that, while the goalkeeper is seeking to anticipate the direction in which their opponent will kick the ball, the kicker is at the same time trying to anticipate the direction in which the goalkeeper will move. For instance, if the goalkeeper were to move toward the left, then a kicker with a goalkeeper-dependent strategy may seek to pick up that information and kick the ball to the right. This interplay is the essence of the interactions that we argue play a vital role in many anticipatory situations, and an experimental paradigm that fails to effectively simulate these situations will fall short of sampling the nature of expertise in many anticipatory tasks.

Conversely, it has become increasingly common for goalkeepers, through their actions, to try to alter the actions of the kicker. Simple actions such as raising the hands [25], waving their hands [26], or even just standing still [27] have been shown to alter where a kicker directs their kick. An interactive paradigm would also be beneficial for other actions such as when the goalkeeper feigns to move in one direction but is able to alter their direction if the kicker commits to one side. Further, there has been recent interest in the effect of a goalkeeper approaching a kicker when placing the ball on the penalty spot in an apparent attempt by the goalkeeper to distract the kicker [28]. Again, an interactive paradigm would be beneficial for exploring the dynamic interplay in scenarios such as these.

The role of interactivity is even important before the actual penalty kick has begun. Contextual or prior information is that which is typically available before the onset of the opponent’s action [29–31] and can also become available or change during the action (e.g., dynamic contextual information) [32, 33]. Contextual information specifies to the observer what type of actions are most probable on the basis of previous behaviors. Penalty situations are increasingly characterized by the provision of contextual information, whereby goalkeepers are provided with information about the prior behavior of opponents, for instance, information about the direction in which each individual kicker has tended to kick their penalties in the past [34, 35]. Studies that have examined the influence of contextual information on anticipatory skill thus far have typically done so using predetermined likelihoods of an action outcome [36]. For instance, a goalkeeper might need to anticipate the kick direction of kickers who have a certain likelihood of kicking in one direction (e.g., 80%; [35]). Yet this fails to account for the interactivity that can underpin the dynamic nature of contextual information. For instance, if a goalkeeper were to constantly dive in one direction, then a kicker is likely to (eventually) kick to the other direction even if they have a preference to do otherwise. Similarly, a kicker might alter their tendency to kick in one direction if a goalkeeper stands off-center in their preferred direction [37, 38]. Again, an interactive paradigm is necessary to provide a genuine representation of these events. Accordingly, the ability to develop a truly interactive paradigm requires not only the ability to manipulate the kinematic actions of opponents, but also to dynamically alter the likelihood of future events on the basis of prior outcomes. VR and AR provide exciting new opportunities to achieve these goals given that the kinematics underlying the movements of avatars can be readily manipulated, and that rules can be put in place for the avatar, in this case the kicker, that can alter the likelihood of their decisions in a controlled manner based on the movements of the observer. Dynamic modeling of real-life situations (e.g., real penalty scenarios) will help in the development of an experimental paradigm capable of representative contextual interactions and the resulting kinematics.

## Advancing the Field Methodologically

It is obvious that effectuating this paradigmatic shift requires resources and interdisciplinary skillsets. A first step would be to use motion capture systems to capture complex dynamic dyadic interactions. Staying with the penalty example, the kinematics of both the penalty taker and goalkeeper could be used to program and model avatars displaying prototypical interactive dynamics. New advances in markerless motion capture make it much easier and affordable to capture the kinematics in a noninvasive way, even in game situations. Subsequently, the VR settings including the scenarios in which the avatars adapt their movements depending on the participants’ motor responses can be modeled. We suggest a modeling approach (though other possibilities are certainly viable) to specify naturalistic, prototypical behaviors and corresponding behavioral changes that will then form the basis of creating both temporally and spatially complex, representative interaction scenarios. It will no doubt be challenging to produce novel kinematic actions that are modified in “real-time,” rather than avatars with predetermined kinematics, but new techniques such as motion matching in the field of animations make this eminently possible. In this way, avatars can respond in highly realistic ways that are dictated by the actions and tendencies of others. The resulting scenarios will then form the experimental foundation to test anticipation in fundamentally new but controlled ways, i.e. when we interact with others rather than when we merely observe them. In addition, going beyond such events may not only include interactions between humans, but also non-human actors such as robots [20].

Notably, research from other approaches such as the ecological dynamics approach [39–41], despite not addressing interactive scenarios from an anticipation perspective per se, have examined tasks and developed methods to capture dynamic interaction scenarios and provide valuable information for the future study of dynamic interactive anticipation. The data generated and results produced by proponents of this approach seem very informative, for instance, to model avatars for the proposed paradigmatic shift. Yet, as alluded to above, the new dynamic interactive anticipation paradigm aims to explain and predict real-life anticipatory behavior. To this end, mechanisms (i.e., cause–effect relationships) need to be identified, and in our opinion, this is best achieved by means of experimentation, and more specifically, systematic experimental manipulation. This will be achieved by applying technologies such as AI, VR, and AR [42], which allow us to maintain full experimental control, meaning that, for instance, each participant will experience identical scenarios. In contrast, this is unachievable by analyzing representative human encounters or interactions as in Vilar et al. [41], due to the fact that each and every movement, and consequently situation, is different (degrees of freedom problem [43]).

## Conclusions

A considerable amount of work has been done to understand the ability of humans to anticipate the actions of others, but we argue that much more remains to be discovered. In particular, the highly constrained paradigms that have been relied on to date have failed to account for the dynamic and emergent interactions that form the basis of many social scenarios. Significant advances are required to develop a genuinely interactive paradigm, in particular advances in the ability to manipulate the kinematic actions of others in real time, and to dynamically alter the likelihood of their action outcomes on the basis of prior outcomes. Fortunately, recent advances in virtual reality, computer animation, and artificial intelligence mean that a truly interactive paradigm will soon be possible. This is true for discrete tasks (e.g., tennis serve) predominantly studied in the past as much as for more continuous and complex tasks (e.g., a three-on-three play in soccer) for which the role of anticipation remains largely unexplored [44]. We hope researchers will take on the challenge of developing appropriate scenarios in an effort to achieve the paradigmatic shift necessary to better understand the true nature of anticipation in human interactions.
